# Blended learning mediated fostering of students’ engagement in an undergraduate medical education module

**DOI:** 10.15694/mep.2019.000127.1

**Published:** 2019-06-10

**Authors:** Lena Jafri, Hafsa Majid, Hassan Salman Siddiqui, Najmul Islam, Faraz Khurshid

**Affiliations:** 1 Aga Khan University

**Keywords:** Student engagement, flipped classroom, Flashcards, Kahoot, crossword puzzles, millennial students

## Abstract

This article was migrated. The article was marked as recommended.

Introduction

The study investigated students’ perception of introducing blended learning strategies to engage and motivate them towards lecture halls and Problem Based Learning (PBL) sessions for improving the quality of the overall students’ learning experience.

Methods

A prospective study was conducted at the Aga Khan University Medical College involving a total of one hundred four (104) year II medical students for the Endocrine and Reproduction module in the academic year 2018. This module ran for six weeks. Modifications in teaching methods were made for this module introduced after approval from Endocrine & Reproduction module, year II and curriculum committees. Written informed consent was obtained from facilitators and students. To assess the effectiveness of modifications introduced, students’ feedback was taken on a Likert’s scale of 1-5, at the end of the module. Data were analyzed by SPSS version 20.

Results

Total 94 (90%) students participated in the written feedback. Forty percent medical students (n=38) responded that videos were useful, 54% (n=51) acknowledged that post-PBL activities like crossword puzzles and Kahoot games, created significant discussions amongst students in a fun yet intellectual manner. Seventy percent of students (n=66) agreed that access to Kahoot was easy and no connectivity issues were faced. Twenty percent students (n=20) endorsed that flipped lectures enhanced understanding of topics, 87% students (n=82) felt that recorded lecture was very helpful and flash cards were found helpful by 14% students (n=12).

Conclusion

Student engagement is an important issue in medical schools in this era. Results showed that blended learning and educational activities enhanced students’ learning motivation and interest.

## Introduction

Student engagement in higher education is considered as a collaborative effort brought forward by learners, teachers, and institutions for quality learning ( Zepke, 2013b). Students are likely to engage if they are supported by teachers who work for an effective learning environment, expect a high standard, challenge and volunteer themselves freely available to discuss student academic progress ( Bryson and Hand, 2007). Increasing student engagement is one way of enhancing the quality of learning and improving knowledge of students ( Zepke, 2013a). An institution is able to influence student engagement in a number of ways, one being through curriculum design ( Akram *et al.*, 2018). Furthermore, millennial students have the ability to control technology, have forthcoming educational experience and higher expectations of managing their ‘learning space’ ( Dziuban, Moskal and Hartman, 2005).

The technology-based approach to student engagement can be reinforced by revolutionary approaches to learning such as blended learning and the flipped classroom. A newer way of engaging students and making them attend lectures attentively is by blended learning ( Pereira *et al.*, 2007; Rehman, Afzal and Kamran, 2013). The Higher Education Academy (HEA) states, ‘there is a huge range of different blended approaches; the balance between online and face-to-face components, and the integration of other methods, depends on the needs of learners and the context within which the learning is implemented’ ( *Blended learning | Higher Education Academy*, 2017). Moreover, a flipped classroom is an instructional strategy that reverses the traditional learning environment by delivering instructional content, often online and outside of the classroom ( Hew and Lo, 2018). It is not a new concept and can be equated with pedagogies such as active learning, peer instruction, case-based or problem-based learning (PBL), or, any blended learning strategy that requires students to prepare to learn before they meet and engage with peers in purposeful activities ( Shah, Walters and McKillop, 2008).

The never-ending strain on faculty involved in the PBL curriculum is to demonstrate its effective delivery with the sustainability of educational benefits for lifelong learning ( Kow *et al.*, 2017). The incorporation of these technology-based methods in PBL can foster student engagement with a high degree of satisfaction, and improved efficiency in students’ performance ( Guerrero, 2017).

We introduced face to face and online activities (blended learning approach) in one of the modules taught in the second year of medical school. Our aim was to engage students in PBL sessions and also to motivate them towards lecture halls to attend lectures. The objective of this study was to understand students’ response to modifications and its impact on the quality of the overall student experience.

## Methods

This prospective study was conducted at Aga Khan University Medical College, Karachi, Pakistan. Modifications in teaching and learning methods were made for the Endocrine and Reproduction module in the academic year 2018 of year II undergraduate medical students. The For PBL sessions, the students were divided into 12 groups, each consisting of eight to nine students. Ten PBL cases were conducted in endocrine and reproduction module which was scheduled for six weeks duration. Approval from the institutional ethical review committee was taken before commencement of the study (ERC ID: 2018-0160-248). The following modifications were introduced after approval from Endocrine Reproduction Module, year 2 and curriculum committees:

• In PBLs cases, activities were introduced ( Table 1).

**Table 1.** Problem Based Learning Cases and the Activities conducted in each case.

PBL Case	Activities Conducted
Cases 1: Acromegaly	Crossword puzzle
Cases 2: Cushing’s Syndrome	Quiz by ‘Kahoot
Case 3: Thyroid and Parathyroid Disorders	Quiz by ‘Kahoot’ A video of hypocalcemia sign
Case 4: Diabetes Mellitus	Flash card Quiz by ‘Kahoot’
Case 5: Congenital Adrenal Hyperplasia	Assignment
Case 6: Male Reproductive System	‘Kahoot’ quiz
Case 7: Genetic Disease	No Activity
Case 8: Menstrual Irregularity	
Case 9: Ovulation, fertilization, pregnancy, and lactation	
Case 10: Infertility	Crossword puzzle

• Flashcards of important concepts related to the topics being taught were uploaded on One45 which is an online software to manage, schedule, assess, and track medical students’ curriculum more effectively.
• Some of the lectures were flipped and educational materials related to the topic being taught were shared with students on One45 before a flipped lecture.
• One lecture was recorded live and recording made available to students after the lecture was delivered.

Facilitators were trained for conducting quizzes, making live video recordings and gaming activities before the introduction of the module. A step by step Kahoot guide was developed for the PBL facilitators and distributed in the facilitator’s meeting. This was reinforced along with user ID and password to access the game a day before the activity as most of the facilitators were new to Kahoot, Consents were taken from facilitators and students at the start of the module. To assess the perception of students and faculty towards these modifications feedback was taken from all facilitators and students at the end of the module.

### Statistical Analysis

Microsoft Excel was used to enter and analyze the data. Descriptive statistics were used to analyze the data. Feedback regarding the integration of blended cases (PBL cases with videos), online activities, access to educational material, flipped classroom teaching and video recorded LCFs was taken on a Likert’s scale of 1-5 (Strongly agree, somewhat agree, neutral, somewhat disagree, strongly disagree). Frequency of responses by the students was generated.

## Results/Analysis

**Figure 1.**
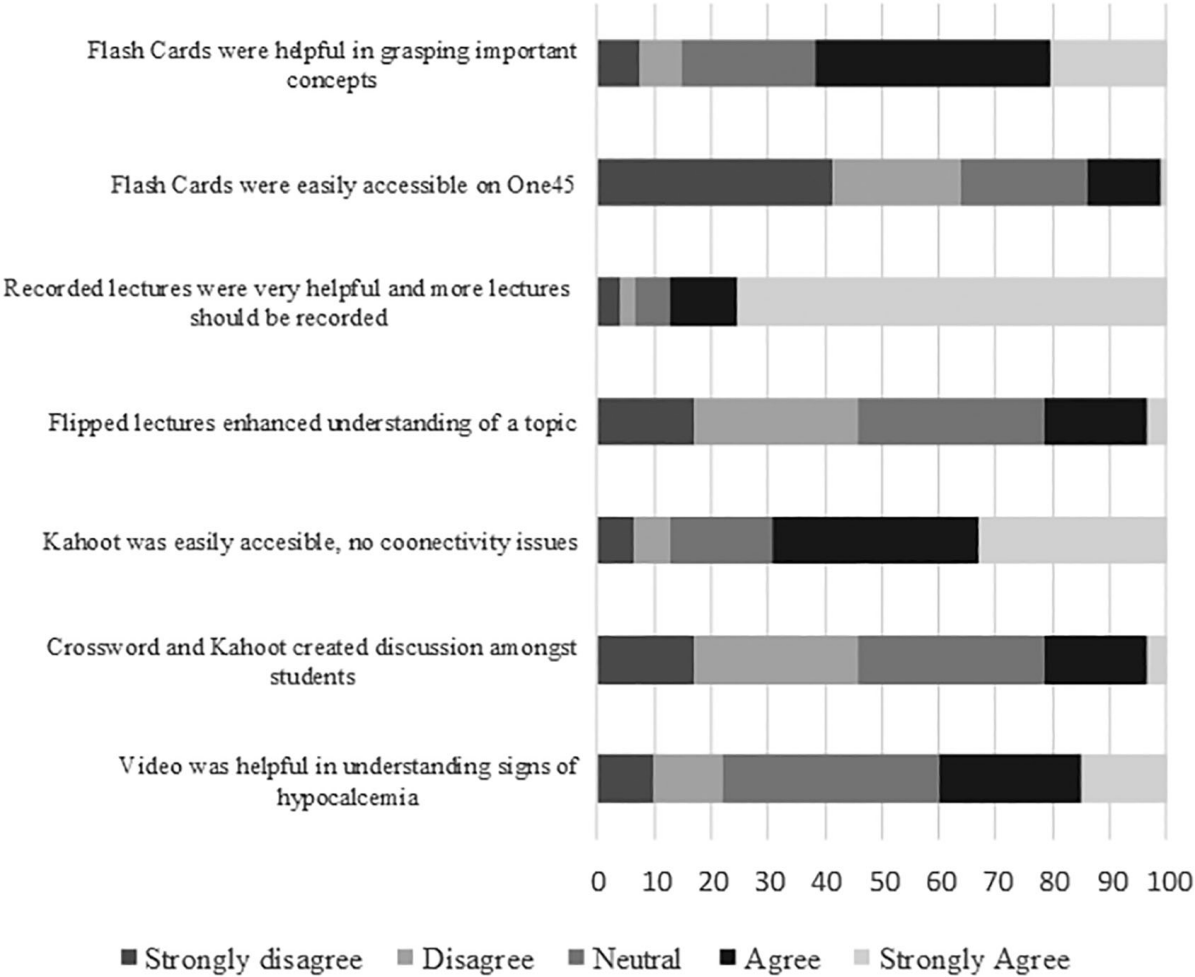
Feedback of Medical Students regarding modifications incorporated into the Endocrine and Reproduction module.

Ninety-four medical students (90%) out of a total of 104 students enrolled in year-II participated in the feedback. In PBL case-2, titled on thyroid-parathyroid disorder a video showing signs of hypocalcemia was introduced for a better understanding of the clinical presentation of hypoparathyroidism patients. Medical students (n=38, 40%) responded that video was helpful in understanding signs of hypocalcemia. At the end of 4 PBL sessions, online quiz activity (using Kahoot) and 2 PBL sessions crossword puzzles were conducted. Students (n=51, 54%) agreed that activities at the end of PBL sessions, like crossword and Kahoot, created discussion amongst students in a fun learning way. Seventy percent of students (n=66) responded that access to Kahoot was easy without any network connectivity issues, shown in figure 1.

Two Large class format (LCF) lectures were flipped and educational materials related to the topic being taught were shared with students two days prior to the lecture. Students (n=20, 21%) agreed that flipped lectures enhanced understanding of the topic. One LCF lecture was recorded live and recording was shared with the students. Students (n=82, 87%) responded that more lectures should be recorded in the future.

Flashcards on 2 topics “Criteria of Diabetes mellitus diagnosis” and “Biochemical findings to differentiate Cushing’s and Addison’s disease” were shared with the students by uploading on One45. Only 14% of students (n=12) were able to access the flashcards and they responded that the flash cards were helpful in grasping important concepts.

## Discussion

Most of the **s**tudents found that the modifications in the module improved their educational experience. One of the interventions included the video lectures (VL), these lectures have become popular in education as technology made these videotapes more affordable and accessible. It is considered as a powerful communication and instructional tool that provide students the control, convenience and flexibility of their learning ( Dale and Pymm, 2009; Ramlogan, Raman and Sweet, 2014). The best response received was for video recorded lecture, the students felt that more of large class format (LCF) lectures should be recorded. Although only 20% of students attended the regular LCFs, 87% of students learned from the video recorded lectures. These findings were consistent with the study conducted by ( Scagnoli, Choo and Tian, 2019), they demonstrated that students’ satisfaction with VL has a strong association with a positive overall learning experience and perception of the influence of video on learning. Moreover, it can improve student engagement with content because of learners’ control of media and instructors ‘presence. On the contrary, the faculty showed some reservations that students will become lazy; some were concerned with the future replacement of all LCFs by recorded lectures. Another issue raised by faculty was poor quality of the recording, a solution brought forward by them was the use of PowerPoint presentation with voice over.

To foster students’ engagement in LCFs and to alleviate faculty concerns regarding recorded lectures an approach that can be undertaken is to use a combination of faculty delivered LCF and video recorded lectures in a module. In the subsequent cycle, the video recording of previous years’ lectures can be provided while video lectures in the last cycle should be updated and presented as LCFs. The recorded lectures should be updated at least after every two years to keep students abreast with the recent practices and if needed, this pool can be utilized for the teaching of allied health professionals as well. As the attention span of a person is reported to be 20 minutes, the live or recorded lectures duration should not be more than 15-20 minutes ( Wilson and Korn, 2007; Bradbury, 2016).

Student engagement in the learning process can be actively facilitated by introducing online quizzes and crossword puzzle activities. A crossword puzzle is considered as an innovative teaching module. This type of activities not only foster learner attitude towards learning but also provide them a recreational break during traditional lecture class, thus positively affecting students’ skill and performances ( Patrick *et al.*, 2018). In order to prepare students for theory examination and involve them in healthy competition, online quizzes and crossword activities were done at the end of the PBL sessions. After the quiz, students discussed the results amongst themselves and feedback was provided by the facilitator. It was suggested by the faculty that such activities stimulated discussion and helped to clarify important concepts. A pool of quizzes can be made and this strategy can be introduced in other modules as well. A similar interventional study done at a medical school in Egypt reported that students who participated in online course quizzes had significantly better results and learning experience compared to those who took the traditional course ( Abdelhai *et al.*, 2012).

To better grasp, the important concepts flashcards related to the topics being taught were shared with students by uploading on One45. The effectiveness of flashcard-based study has been established, the findings propose that this teaching strategy can help students to achieve a deeper level of knowledge processing such as comprehension and application in a self-directed way which eventually improves student’s performance ( Senzaki *et al.*, 2017). However, the current study showed that most of the students find flashcards ineffective; the major reason identified was the lack of easy accessibility of flashcards on One45. All students and faculty were given limited access to it. Furthermore, the students felt that educational materials of the module uploaded on this software should be shared directly via email so they can access it easily on their cellphones. Two LCFs were flipped to increase interest and engagement of students but again students faced problems in retrieving the educational materials on One45.

Student’s interest and engagement in lectures and PBLs is a major issue faced by the faculty in medical colleges nowadays. Taking into account the changes in the digital world over the last decades, students of the current generation expect technology to be used in advancing their learning, a need to change traditional passive learning methodologies to an active multisensory experimental learning methodology. Students nowadays are wary of classroom teaching, thus there is a need to develop blended curriculum to stimulate student’s interest and improve their learning experiences.

## Conclusion

This study highlights few technologies assisted modifications in the curriculum which were found useful in improving the learning experience of medical students. Such modifications increase students’ engagement and stimulate active discussion. Millennial students have forthcoming educational experience and higher expectations of managing their ‘learning space’. These modifications can be used in other modules as well to improve their learning experience.

## Take Home Messages

Given the changes in the digital world over the last decades, students of the current generation expect technology to be used in advancing their learning, requiring a transition from traditional passive learning methodologies to an active multisensory experimental learning methodology. Blended learning approach can engage students and motivate them towards lecture halls, PBL sessions and contribute to improving the quality of the overall student experience. Other ways which can increase students engagement is asynchronous teaching, using video-recorded lectures, and flashcards.

## Notes On Contributors

**Dr. Lena Jafri** is an Assistant Professor and Residency Director at Aga Khan University Karachi. Being a Consultant Chemical Pathologist she has formal qualifications and clinical research interests in biomarkers of inherited metabolic diseases and musculoskeletal medicine. She is a FAIMER fellow and has been actively involved in the Undergraduate teaching and other educational activities of the institute.

**Dr. Hafsa Majid** (MBBS, FCPS Chemical Pathology) is currently working as a senior instructor, at Departments of Pathology & Laboratory Medicine, Aga Khan University, Karachi. She has been involved in undergraduate and postgraduate teaching for 5 years.

**Dr. Hasan Salman Siddiqi** (MBBS, Ph.D.)Assistant Professor Pharmacology, Department of Biological and Biomedical Sciences Aga Khan University Medical College,Karachi, has an experience of over 18 years in undergraduate and postgraduate medical education. He has a grant and a number of publications to his credit in educational research.

**Dr. Najmul Islam** Graduate of Dow Medical College, Karachi; Post-graduation in Internal Medicine from the UK. Started career from Aga Khan University and presently a Professor for almost 14 years. He established Pakistan Endocrine Society and was one of its past presidents. Served undergraduate education in various capacities and presently Director Undergraduate Medical Education in the Dept of Medicine. Published more than 75 papers in peer-reviewed indexed journals. Delivered more the 225 invited lectures at national and international conferences

**Dr. Faraz Khurshid** (MBBS, MS Molecular Biology, MHPE) is currently working as a senior instructor, joint appointee at Departments of Educational Development and Pathology & Laboratory Medicine, Aga Khan University, Karachi. His enduring relationship with academics and relevant research skills encompasses from basic sciences, molecular biology, to health professions education. His research interests include metacognition, threshold concepts, and learning environment.
