# Supplementary material for: Blended learning mediated fostering of students’ engagement in an undergraduate medical education module
Source: MedEdPublish (2016). 2019 Jun 10;8:127. [Version 1] doi: 10.15694/mep.2019.000127.1 (PMC10712470; doi:10.15694/mep.2019.000127.1)
Supplement: Peer review history

# Reviewer report: Reviewer response for version 1

**DOI:** 10.21956/mep.19856.r29976

**Published:** 2019-09-20

**Authors:** Stella Briggs

**Affiliations:** 1 University of Buraimi

This review has been migrated. The reviewer awarded 5 stars out of 5 This is an interesting and informative article about blended learning. The study seeks to engage and motivate students in problem-based learning sessions. We are in the digital world where daily interactive activities (including education) are now done online extensively. Medical educators need to be creative in capturing students’ interest in learning. This article has given a good insight into few possibilities in the area of fostering student engagement in the classroom, an effort that will that ignite dormant creative skills of some medical educators in teaching.

Reviewer Expertise:

NA

No decision status is available

# Reviewer report: Reviewer response for version 1

**DOI:** 10.21956/mep.19856.r29977

**Published:** 2019-07-12

**Authors:** Trevor Gibbs

**Affiliations:** 1 AMEE

This review has been migrated. The reviewer awarded 3 stars out of 5 An interesting paper that provided some interesting ideas and certainly matched the title of blended learning.My worry with this and these types of papers is that we need to look towards a better evaluation model that one which is based upon self-perception- we need to look at the longer term effects.

Reviewer Expertise:

NA

No decision status is available

# Reviewer report: Reviewer response for version 1

**DOI:** 10.21956/mep.19856.r29980

**Published:** 2019-06-25

**Authors:** Priyanka Daniel

**Affiliations:** 1 Christian Medical College Vellore

This review has been migrated. The reviewer awarded 5 stars out of 5 The article brings out a new concept of blending PBL with blended learning methodologies. The innovative use of technology in constructing a better learner experience is appreciated. The readers are surely benefitted by the work done by the author and their team. A great effort!

Reviewer Expertise:

NA

No decision status is available

# Reviewer report: Reviewer response for version 1

**DOI:** 10.21956/mep.19856.r29981

**Published:** 2019-06-24

**Authors:** Wunna Tun

**Affiliations:** 1 Myanmar Medical Association

This review has been migrated. The reviewer awarded 4 stars out of 5 I found the piece well written, interesting, and likely to help inform the practice of others about Blended learning. The authors pointed out the effective use of audio visual and flash card in teaching and learning for 21st century medical students. Understood well by the medical educationist. I recommend this article as a useful contribution to the field that should be read by those with an interest in the area.

Reviewer Expertise:

NA

No decision status is available

# Reviewer report: Reviewer response for version 1

**DOI:** 10.21956/mep.19856.r29978

**Published:** 2019-06-11

**Authors:** Sibtain Ahmed

**Affiliations:** 1 The Aga Khan university

This review has been migrated. The reviewer awarded 4 stars out of 5 A good read. The author's have highlighted the transformative potential of blended learning in the context of the challenges facing higher education, specially in resource constrained setups. The ability to electronically support modular teaching at virtually no cost is clearly a model that will be adopted widely.

Reviewer Expertise:

NA

No decision status is available

# Reviewer report: Reviewer response for version 1

**DOI:** 10.21956/mep.19856.r29979

**Published:** 2019-06-10

**Authors:** Felix Silwimba

**Affiliations:** 1 University of Lusaka

This review has been migrated. The reviewer awarded 5 stars out of 5 this is an informative article on classroom learning for the Millennial learners. I agree they are wary of classroom learning. I have picked a lot of points. although some may not be applicable in some some parts of Low middle Income countries. I have experimented with student reflection notes of student diary keeping that I checked before the next lesson or at the end of the module. I would like to learn from others.

Reviewer Expertise:

NA

No decision status is available
